# C-Reactive Protein Levels Are Associated with Complement C4 Deposits and Interstitial Arteritis in ANCA-Associated Renal Vasculitis

**DOI:** 10.3390/ijms24043072

**Published:** 2023-02-04

**Authors:** Peter Korsten, Eva Baier, Samy Hakroush, Björn Tampe

**Affiliations:** 1 Department of Nephrology and Rheumatology, University Medical Center Göttingen, 37075 Göttingen, Germany; 2 Institute of Pathology, University Medical Center Göttingen, 37075 Göttingen, Germany; 3 SYNLAB Pathology Hannover, SYNLAB Holding Germany, 86156 Augsburg, Germany

**Keywords:** C-reactive protein, CRP, ANCA-associated vasculitis, MPO-ANCA, PR3-ANCA, interstitial arteritis, complement system, complement C4 deposition

## Abstract

Anti-neutrophil cytoplasmic antibody (ANCA)-associated vasculitis (AAV) is a potentially life-threatening systemic small-vessel vasculitis that is characterized by pauci-immune glomerulonephritis in case of kidney involvement, representing a major denominator of AAV mortality. Innate immunity with complement system activation is increasingly recognized in the pathogenesis of AAV and as an attractive therapeutic target. Although C-reactive protein (CRP) was thought to be a passive, nonspecific marker of inflammation, recent studies indicate that CRP plays a key role in the innate immune system by recognizing pathogens and altered self-determinants. Elevated baseline CRP at disease onset of AAV has already been described as a determinant of poor long-term outcomes. However, its clinical implications at disease onset of AAV, with respect to vasculitis manifestations and complement system activation that might also affect long-term outcomes, remain elusive. CRP levels were retrospectively analyzed in 53 kidney-biopsy-confirmed cases of ANCA-associated renal vasculitis; a total of 138 disease controls were also evaluated. Univariate and multivariate regression analysis was performed on clinicopathological parameters associated with CRP levels in ANCA-associated renal vasculitis. Results: Compared to disease controls, CRP elevation was common in ANCA-associated renal vasculitis and associated with de novo disease (*p* = 0.0169), critical illness (*p* = 0.0346), and severe deterioration of kidney function (*p* = 0.0167), independent of extrarenal disease manifestations. As confirmed by multiple regression analysis, CRP levels were correlated with active lesions predominated by interstitial arteritis in renal vasculitis, specifically with MPO-ANCA seropositivity (*p* = 0.0017). Based on analysis of systemic complement system activation and intrarenal complement deposits, CRP elevation was correlated specifically with complement C4 deposits in interstitial arteries in the subgroup with myeloperoxidase (MPO)-ANCA seropositivity (*p =* 0.039). Finally, this association was independent of systemic complement system activation, as reflected by the consumption of respective complement components. Here, we expand our current understanding of CRP in ANCA-associated renal vasculitis not only as an inflammatory marker, but potentially also as being involved in the pathogenesis of kidney injury by interaction with the complement system.

## 1. Introduction

Anti-neutrophil cytoplasmic antibody (ANCA)-associated vasculitis (AAV) is a potentially life-threatening systemic small-vessel vasculitis that is characterized by pauci-immune glomerulonephritis in case of kidney involvement, representing a major denominator of AAV mortality [1,2,3,4]. Two principal antigens on neutrophils—namely, proteinase 3 (PR3) and myeloperoxidase (MPO)—provide epitopes for ANCA binding, promoting endothelial damage and vascular inflammation, culminating in necrotizing vasculitis [5,6,7]. Innate immunity with complement system activation is increasingly recognized in the pathogenesis of AAV and as an attractive therapeutic target [8,9]. Although C-reactive protein (CRP) was thought to be a passive, nonspecific marker of inflammation, recent studies indicate that CRP plays a key role in the innate immune system by recognizing pathogens and altered self-determinants [10,11]. CRP is synthesized by the liver in response to pro-inflammatory cytokines during acute inflammation and is capable of activating the classical pathway of the complement system [12,13,14]. CRP primarily binds to phosphocholine-containing molecules, and CRP–phosphocholine complexes activate the classical pathway of the complement system [15,16,17,18,19,20]. Elevated baseline CRP at disease onset of AAV has already been described as a determinant of poor long-term outcomes [21,22,23,24]. However, its clinical implications at disease onset of AAV, with respect to vasculitis manifestations and complement system activation that might also affect long-term outcomes, remain elusive. Therefore, in this study, we aimed to systematically describe clinicopathological findings associated with baseline CRP levels at disease onset of AAV in kidney-biopsy-confirmed severe ANCA-associated renal vasculitis.

## 2. Results

### 2.1. CRP Elevation Was Associated with Active ANCA-Associated Renal Vasculitis Independent of Extrarenal Disease Manifestations

**Figure 1:**
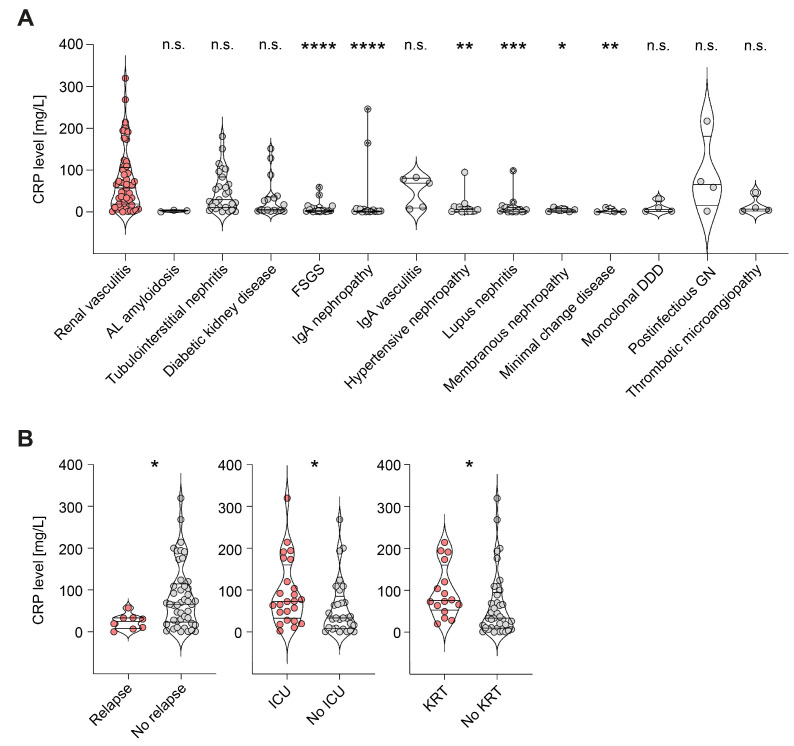
CRP elevation is associated with active ANCA-associated renal vasculitis: (**A**) Violin plots and individual data points for CRP levels are shown in renal vasculitis (n = 53) and disease controls, including AL amyloidosis (n = 3), tubulointerstitial nephritis (n = 27), diabetic kidney disease (n = 17), FSGS (n = 18), IgA nephropathy (n = 17), IgA vasculitis (n = 5), hypertensive nephropathy (n = 13), lupus nephritis (n = 16), membranous nephropathy (n = 6), minimal change disease (n = 4), monoclonal DDD (n = 4), post-infectious GN (n = 4), and thrombotic microangiopathy (n = 4). The Kruskal–Wallis test with Dunn’s multiple correction was performed for group comparisons (n.s. = not significant, * *p* < 0.05, ** *p* < 0.01, *** *p* < 0.001, **** *p* < 0.0001). (**B**) CRP levels after group separations according to relapsing disease, requirement of ICU supportive care, and KRT. Group comparisons were performed with the Mann–Whitney *U*-test (* *p* < 0.05). Abbreviations: ANCA, anti-neutrophil cytoplasmic antibody; CRP, C-reactive protein; FSGS, focal segmental glomerulosclerosis; IgA, immunoglobulin A; DDD, dense deposit disease; GN, glomerulonephritis; ICU, intensive care unit; KRT, kidney replacement therapy.

We first compared CRP levels at admission in a total of 53 patients with kidney-biopsy-confirmed ANCA-associated renal vasculitis, along with 138 disease controls. CRP levels were significantly elevated in renal vasculitis compared to focal segmental glomerulosclerosis (FSGS, *p* < 0.0001), immunoglobulin A (IgA) nephropathy (*p* < 0.0001), hypertensive nephropathy (*p* = 0.0025), lupus nephritis (*p* = 0.0003), membranous nephropathy (*p* = 0.0231), and minimal change disease (*p* = 0.0067, Figure 1A). In contrast, AL amyloidosis (*p* = 0.0588), tubulointerstitial nephritis (*p* > 0.9999), diabetic kidney disease (*p* = 0.2425), IgA vasculitis (*p* > 0.9999), monoclonal dense deposit disease (DDD, *p* = 0.4730), post-infectious glomerulonephritis (GN, *p* > 0.9999), and thrombotic microangiopathy (*p* > 0.9999) presented CRP elevation that was not significantly different from that of renal vasculitis (Figure 1A). The relevance of CRP levels was confirmed by elevation in de novo disease (*p* = 0.0169), critical illness with requirement of intensive care unit treatment (*p* = 0.0346), and severe deterioration of kidney function requiring kidney replacement therapy (KRT, *p* = 0.0167, Figure 1B). Interestingly, there were no significant differences in CRP levels with respect to extrarenal AAV manifestations (Table 1), indicating that CRP elevation was predominantly correlated with kidney involvement. In summary, CRP elevation was common in ANCA-associated renal vasculitis and associated with de novo disease, critical illness, and severe deterioration of kidney function, independent of extrarenal disease manifestations.

### 2.2. CRP Levels Were Correlated with Active Glomerular and Tubulointerstitial Renal Vasculitis with MPO-ANCA Seropositivity

**Figure 2:**
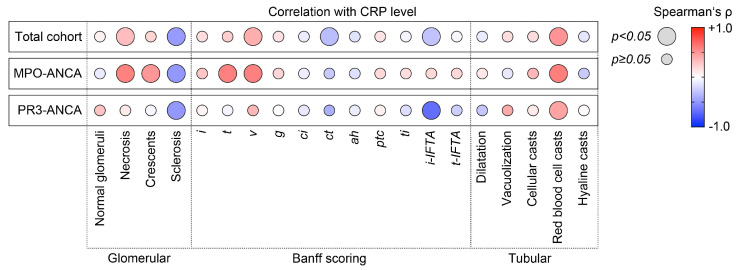
CRP elevation is correlated with complement C4 deposits in interstitial arteries in MPO-ANCA-associated renal vasculitis. Correlations between CRP levels, serum C3 and C4 measurements, and intrarenal complement C3 and C4 deposits localized to different vascular compartments in ANCA-associated renal vasculitis are shown by heatmaps reflecting the mean values of Spearman’s ρ; circle size represents the significance level.

Based on our observation that elevated levels of CRP were correlated with deterioration of kidney function, we next analyzed active and chronic intrarenal lesions in association with CRP levels in ANCA-associated renal vasculitis. In the total cohort, CRP levels were positively correlated with glomerular necrosis (Spearman’s ρ = +0.32, *p* = 0.0212), interstitial arteritis v (ρ = +0.39, *p* = 0.0111), and red blood cell casts (ρ = +0.50, *p* = 0.0003, Figure 2). Interestingly, this association between CRP levels and active glomerular and tubulointerstitial renal vasculitis was only detectable in the subgroup with MPO-ANCA seropositivity (glomerular necrosis: ρ = +0.58, *p* = 0.002, crescents: ρ = +0.49, *p* = 0.0103, tubulitis t: ρ = +0.59, *p* = 0.002, interstitial arteritis v: ρ = +0.59, *p* = 0.0067, red blood cell casts: ρ = +0.59, *p* = 0.0018, Figure 2). Multiple regression analysis confirmed that interstitial arteritis v was the predominant variable associated with levels of CRP in MPO-ANCA-associated renal vasculitis (ß = +0.66, *p* = 0.0017, Table 2). Except for red blood cell casts (ρ = +0.43, *p* = 0.0429), none of these lesions were correlated with levels of CRP in the subgroup with PR3-ANCA seropositivity (Figure 2). In summary, CRP levels were correlated with active lesions predominated by interstitial arteritis in renal vasculitis, specifically with MPO-ANCA seropositivity.

### 2.3. CRP Elevation Was Correlated with Complement C4 Deposits in Interstitial Arteries in MPO-ANCA-Associated Renal Vasculitis

**Figure 3:**
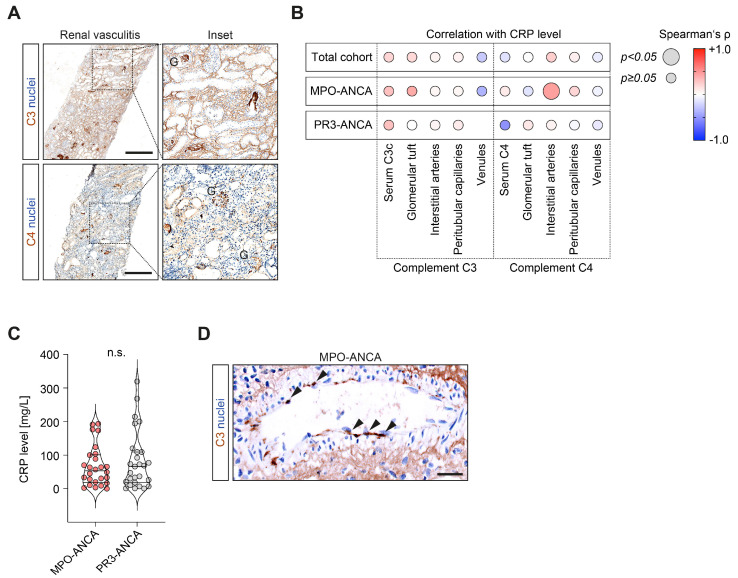
CRP elevation is correlated with complement C4 deposits in interstitial arteries in MPO-ANCA-associated renal vasculitis: (**A**) Immunostainings for intrarenal complement C3 and C4 deposits, with insets; positive glomeruli (G) are indicated (scale bars: 500 µm). (**B**) Correlations between CRP levels, serum C3 and C4 measurements, and intrarenal complement C3 and C4 deposits localized to different vascular compartments in ANCA-associated renal vasculitis, as shown by heatmaps reflecting the mean values of Spearman’s ρ; circle size represents the significance level. (**C**) CRP levels after group separations according to ANCA subtypes. Group comparisons were performed with the Mann–Whitney *U*-test (n.s. = not significant). (**D**) Representative immunostaining for intrarenal complement C4 deposits in interstitial arteries in the subgroup with MPO-ANCA seropositivity; arrowheads indicate endothelial cells positive for complement C4 (scale bar: 25 µm). Abbreviations: ANCA, anti-neutrophil cytoplasmic antibody; CRP, C-reactive protein; G, glomerulus; MPO, myeloperoxidase; PR3, proteinase 3.

Based on our observation that elevated CRP levels were correlated with active glomerular and tubulointerstitial renal vasculitis, we finally analyzed their association with systemic complement system activation and intrarenal complement deposits (Figure 3A). We did not observe any association between CRP levels and systemic complement system activation reflected by serum C3c and C4 measurements (Figure 3B). Among intrarenal complement deposits, the only significant correlation was observed between CRP elevation and complement C4 deposits in interstitial arteries, specifically in the subgroup with MPO-ANCA seropositivity (ρ = +0.45, *p* = 0.039, Figure 3B,C). This observation was not attributed to significant differences in complement C4 deposits between MPO-ANCA and PR3-ANCA seropositivity per se (Table 3). In summary, CRP elevation was correlated with interstitial arteritis along with complement C4 deposits in interstitial arteries, specifically in the subgroup with MPO-ANCA seropositivity. Furthermore, this association was independent of systemic complement system activation, as reflected by the consumption of respective complement components.

## 3. Discussion

CRP is part of the innate immunity involved in nonspecific opsonization and neutralization of antigens, in contrast to the antigen-specific adaptive/acquired immunity maintained by antibodies [25]. While elevated baseline CRP at disease onset of AAV has already been described as a determinant of poor long-term outcomes, its clinical implications at disease onset of AAV with respect to vasculitis manifestations and complement system activation remain elusive [21,22,23,24]. Therefore, in this study, we aimed to systematically describe clinicopathological findings in association with baseline CRP levels at disease onset of AAV in kidney-biopsy-confirmed severe ANCA-associated renal vasculitis. Baseline CRP elevation was common in ANCA-associated renal vasculitis and associated with de novo disease, critical illness, and severe deterioration of kidney function. This is especially relevant because a higher initial mortality rate of critically ill patients within the first hospital stay was observed, while the long-term mortality of hospital survivors did not differ between the ICU and non-ICU groups [26]. Regarding the kidneys, severe renal dysfunction has already been associated with poor long-term outcomes in ANCA-associated renal vasculitis [2]. Our observation that we did not find any association between CRP levels at AAV onset and extrarenal disease manifestations further supports the predominant role of CRP levels in severe kidney involvement. Furthermore, we found that CRP levels were correlated with active lesions predominated by interstitial arteritis in renal vasculitis, specifically with MPO-ANCA seropositivity. We and others have recently described how interstitial arteritis represents a subtype of ANCA-associated renal vasculitis affecting a considerable subset of patients [27,28]. In addition, the presence of interstitial arteritis was associated with poor long-term renal outcomes and significantly increased the risk prediction by established scoring systems [28,29]. Interestingly, our observations confirm those of previous reports that the presence of interstitial arteritis was correlated with higher levels of CRP [28]. Our observation that CRP elevation was also correlated with complement C4 deposits in interstitial arteries could therefore further improve our current understanding of the underlying mechanisms contributing to interstitial arteritis in ANCA-associated renal vasculitis.

Regarding the complement system, CRP can bind to the C1q globular recognition domain to activate the classical complement pathway, as well as to ficolins, mannose-binding lectins, and factor H to regulate the alternative and lectin-dependent complement pathways [25]. In kidney allografts, previous studies suggested that CRP can mediate complement activation in vivo [30]. In terms of distinct complement components, C3 is critical for activation of the complement system as a whole, while C4 is the major protein of the classical cascade [31]. Anaphylatoxin C3a stimulates inflammation by inducing an oxidative burst in macrophages, eosinophils, and neutrophils [32,33,34]. Furthermore, C3a and C5a directly activate basophils and mast cells, resulting in histamine production [35,36]. Although the pro-inflammatory effects of C3a are not in question, studies have also highlighted the anti-inflammatory role of C3a in different contexts [37]. Migration of neutrophils and degranulation are prevented in the presence of anaphylatoxin C3a, regardless of whether other granulocytes are activated by C3a [38,39]. The C4 activation product C4a has also been shown to exert a functional activity on macrophages and monocytes [40,41]. Because no C4a receptor has yet been reported, the physiological role of anaphylatoxin C4a and its contribution to autoimmune diseases such as AAV remain elusive [42]. On a mechanistic level, the ability of CRP to activate the complement system is by binding to an appropriate ligand, such as complement C4 [12,19]. We found that this association between CRP elevation, interstitial arteritis, and corresponding complement C4 deposits was specifically present in the subgroup with MPO-ANCA seropositivity. In this context, specific binding of CRP to MPO (and not PR3) has already been described and experimentally linked to complement system activation [43]. In addition, CRP is capable of stimulating the release of MPO in vitro and in vivo [44]. These findings might indicate a pathomechanistic link between MPO seropositivity and CRP in ANCA-associated renal vasculitis. The alternative pathway plays a predominant role in AAV pathogenesis, and it has already been reported that alternative pathway activation of the complement system requires the presence of C4 [45,46,47,48]. We have previously shown that intrarenal complement deposits were not associated with corresponding consumption of serum levels, supporting the concept of intrarenal synthesis of distinct complement system components [49,50]. Therefore, this study expands our current understanding of CRP in ANCA-associated renal vasculitis not only as an inflammatory marker, but also as potentially being involved in the pathogenesis of kidney injury by interaction with the complement system, and possibly local complement synthesis in the kidney. This is especially relevant because targeted therapies to block complement system activation in AAV are currently emerging.

Our study has several limitations, such as the small number of patients and the retrospective study design. Furthermore, our observations are associative and do not prove causality, which would require mechanistic studies. Nevertheless, this study provides a clinically approached link between CRP elevation, complement C4 deposits, and interstitial arteritis limited to MPO-ANCA-associated renal vasculitis, thereby broadening our current pathophysiological understanding.

## 4. Materials and Methods

### 4.1. Study Population and Subgroup Formation

A cohort of 53 kidney-biopsy-confirmed cases of ANCA-associated renal vasculitis was retrospectively enrolled between 2015 and 2020 in a single-center observational study at the University Medical Center Göttingen, Göttingen, Germany [51,52,53,54,55]. In addition, a total of 138 disease controls—including AL amyloidosis, tubulointerstitial nephritis, diabetic kidney disease, focal segmental glomerulosclerosis (FSGS), immunoglobulin A (IgA) nephropathy, IgA vasculitis, hypertensive nephropathy, lupus nephritis, membranous nephropathy, minimal change disease, monoclonal dense deposit disease (DDD), post-infectious glomerulonephritis (GN), and thrombotic microangiopathy—were also evaluated. While no formal approval was required for the use of routine clinical data, a favorable ethical opinion was granted by the local ethics committee (no. 22 February 2014 and 28 September 2017). All participants provided their written informed consent for the utilization of routinely collected data for research purposes as part of their regular medical care. Medical records were used to collect data on age, sex, medication, comorbidities, and laboratory findings, including baseline CRP at the time of kidney biopsy.

### 4.2. ANCA Autoantibody and Complement Measurements

MPO-ANCA (reference range: <3.5 IU/mL) and PR3-ANCA autoantibodies (reference range: <2 IU/mL) were measured by immunoassay (ImmunoCAP 250, Thermo Fisher Scientific, Waltham, MA, USA). Plasma concentrations of human complement components C3c (9D9621, Abbott, Chicago, IL, USA) and C4 (9D9721, Abbott, Chicago, IL, USA) were determined by turbidimetric measurements on the ARCHITECT-C module. Reference range plasma concentrations for circulating C3c and C4 were defined at 0.82–1.93 g/L and 0.15–0.57 g/L, respectively.

### 4.3. Renal Histopathology

A renal pathologist evaluated all kidney biopsies and was blinded to the clinical data analysis. Based on the current version of the Banff scoring system for renal allograft pathology, tubulointerstitial lesions were scored as previously reported: arteriolar hyalinosis (*ah*), arteritis (*v*), glomerulitis (*g*), inflammation in areas of interstitial fibrosis and tubular atrophy (*i-IFTA*), interstitial fibrosis (*ci*), interstitial inflammation (*i*), peritubular capillaritis (*ptc*), total inflammation (*ti*), tubular atrophy (*ct*), tubulitis (*t*), and tubulitis in areas of interstitial fibrosis and tubular atrophy (*t-IFTA*) [56,57]. Tubular injury lesions were systematically assessed as recently described in [58]. Briefly, tubular dilation, tubular necrosis, epithelial simplification, non-isometric cell vacuolization, and red blood cell (RBC) and necrotic casts were scored in a range from 0 to 4, depending on the fraction of affected cortical area of renal biopsy (score 0: <1%, 1: ≥1–10%, 2: ≥10–25%, 3: ≥25–50%, 4: >50%). Moreover, all injured glomeruli (crescentic and/or necrotic) were screened for the presence of a Bowman’s capsule rupture, the extent of which was further quantified as previously described in [59,60,61].

### 4.4. C3c and C4d Immunohistochemistry

Formalin-fixed, paraffin-embedded kidney sections were deparaffinized in xylene and rehydrated in ethanol containing distilled water. Tissue sections were stained using antibodies against C3c (1:10,000, A0062, Agilent Dako, Santa Clara, CA, USA) and C4d (1:50, 503-17344, Zytomed, Berlin, Germany), and labeling was performed using the Novolink^TM^ Polymer Detection System (Leica Biosystems, Wetzlar, Germany) according to the manufacturer’s protocol. Nuclear counterstaining was performed by using Mayer’s Hematoxylin Solution (Sigma, St. Louis, MO, USA). As previously described, kidney biopsies were evaluated for the presence/absence of C3c and C4d deposits in the glomerular tuft, interlobular arteries, peritubular capillaries, and venules [49,50].

### 4.5. Statistical Analysis

Normal distribution was evaluated using the Shapiro–Wilk test; statistical comparisons were not formally powered or pre-specified. Probability values (*p*-values) below 0.05 were considered to be statistically significant. Parameters are shown as the median and interquartile range (IQR); median comparisons were performed with the Mann–Whitney U-test. Heatmaps reflect the mean values of Spearman’s ρ in the univariate linear regression analysis; circle size represents the significance level. For stepwise multiple linear regression, covariates were retained to significant differences in the linear regression model to avoid model overfitting. Data analyses were performed using GraphPad Prism (version 9.4.1 for MacOS, GraphPad Software, San Diego, CA, USA) and IBM SPSS Statistics (version 28.0.1.1 for MacOS, IBM Corporation, Armonk, NY, USA).

## 5. Conclusions

This study expands our current understanding of CRP in ANCA-associated renal vasculitis not only as an inflammatory marker, but also as potentially being involved in the pathogenesis of kidney injury by interaction with the complement system.

## Figures and Tables

**Table 1:** CRP levels after group separation by extrarenal manifestations of AAV.

Clinical Data	No. of Patients	Manifestation	No. of Manifestation	*p*-Value
Lung—CRP (mg/L)	31	33.4 (18.3–109.5)	63.7 (17.7–105.3)	0.9608
Sinus—CRP (mg/L)	9	68.9 (5.1–109.4)	53.4 (20.1–103.1)	0.9880
Joint—CRP (mg/L)	12	65 (34.3–104.2)	49.3 (13.6–106.8)	0.3847
Ear—CRP (mg/L)	4	55.3 (0.6–177.4)	57.4 (20.2–102.1)	0.7070
Eye—CRP (mg/L)	2	191.3 (20.5–268.5)	53.4 (17.8–101.2)	0.2222
Nerve—CRP (mg/L)	6	82.5 (26.7–132)	49.3 (18.3–104)	0.4189
Skin—CRP (mg/L)	9	63.8 (28.3–71.4)	53.4 (16.9–109.5)	0.9138

Medians (IQR) are shown; group comparisons were performed with the Mann–Whitney U-test. Abbreviations: AAV, ANCA-associated vasculitis; ANCA, anti-neutrophil cytoplasmic antibody; CRP, C-reactive protein; No., number.

**Table 2:** Multiple linear regression analyses correlating with CRP levels in MPO-ANCA-associated renal vasculitis.

Variable	β	*p*-Value
Glomerular necrosis—% of total	+0.36	0.0460
Glomerular crescents—% of total	+0.39	0.0229
Tubulitis t—score	+0.29	0.1828
Interstitial arteritis v—score	+0.66	0.0017
Red blood cell casts—score	+0.45	0.0091

Abbreviations: CRP, C-reactive protein.

**Table 3:** Complement C3 and C4 deposits in ANCA-associated renal vasculitis separated by MPO-ANCA and PR3-ANCA seropositivity.

**Complement C3**	**MPO-ANCA**	**PR3-ANCA**	***p*-Value**
Glomerular tuft—% of total	17/26 (65.4)	20/27 (74.1)	0.4909
Interstitial arteries—% of total	1/26 (3.8)	2/27 (7.4)	0.5749
Peritubular capillaries—% of total	17/26 (65.4)	17/27 (63)	0.8542
Venules—% of total	1/26 (3.8)	0/27 (0)	0.3036
**Complement C4**	**MPO-ANCA**	**PR3-ANCA**	***p*-Value**
Glomerular tuft—% of total	17/26 (65.4)	16/27 (22.2)	0.6456
Interstitial arteries—% of total	8/26 (30.8)	12/27 (44.4)	0.3045
Peritubular capillaries—% of total	14/26 (53.8)	13/27 (48.1)	0.6783
Venules—% of total	10/26 (38.5)	6/27 (22.2)	0.1980

Abbreviations: ANCA, anti-neutrophil cytoplasmic antibody; MPO, myeloperoxidase; PR3, proteinase 3.

## Data Availability

The original contributions presented in this study are included in the article; further data and materials are available from the corresponding author upon reasonable request.
